# Development of P*grac*100-based expression vectors allowing high protein production levels in *Bacillus subtilis* and relatively low basal expression in *Escherichia coli*

**DOI:** 10.1186/s12934-015-0255-z

**Published:** 2015-05-21

**Authors:** Trang Thi Phuong Phan, Linh Thuoc Tran, Wolfgang Schumann, Hoang Duc Nguyen

**Affiliations:** Center for Bioscience and Biotechnology, VNUHCMC-University of Science, 227 Nguyen Van Cu District 5, Hochiminh, Vietnam; Laboratory of Molecular Biotechnology, VNUHCMC-University of Science, 227 Nguyen Van Cu District 5, Hochiminh, Vietnam; Department of Microbiology, VNUHCMC-University of Science, 227 Nguyen Van Cu District 5, Hochiminh, Vietnam; Institute of Genetics, Universität of Bayreuth, Bayreuth, D-95440 Germany

**Keywords:** *Bacillus subtilis* 1012, IPTG-inducible promoter, P*grac*, P*grac*100, pHT253, pHT254, pHT255

## Abstract

**Background:**

In general, fusion of recombinant genes to strong inducible promoters allowing intracellular expression in *Bacillus subtilis* is a two-step process. The ligation products are transformed into *Escherichia coli*, followed by identification of the correct plasmid, and this plasmid is subsequently transformed into *B. subtilis*. This raises the problem that basal level of expression of the recombinant gene could be harmful for *E. coli* cells. Based on the P*grac* promoter, we optimized the UP element, the -35, 15, -10 and the +1 region to enhance the promoter activity in *B. subtilis* after induction. However, detailed investigations for a promoter to develop expression vectors that allows high protein production levels in *B. subtilis* and a relatively low basal expression levels in *E. coli* has not been studied yet.

**Results:**

We screened the previously constructed library of *E. coli* – *B. subtilis* shuttle vectors for high level expression in *B. subtilis* and low basal level in *E. coli*. Promoter P*grac100* turned out to meet these criteria, in which ß-galactosidase expression level of P*grac100*-*bgaB* is about 9.2 times higher than P*grac01*-*bgaB* in *B. subtilis* and the ratio of those in induced *B. subtilis* over un-induced *E. coli* from P*grac100*-*bgaB* is 1.3 times higher than P*grac01*-*bgaB*. Similarly, GFP expression level of P*grac100*-*gfp* is about 27 times higher than that of P*grac01*-*gfp* and the ratio from P*grac100*-*gfp* is 35.5 times higher than P*grac01*-*gfp*. This promoter was used as a basis for the construction of three novel vectors, pHT253 (His-tag-MCS), pHT254 (MCS-His-tag) and pHT255 (MCS-Strep-tag). Expression of the reporter proteins BgaB and GFP using these expression vectors in *B. subtilis* at a low IPTG concentration were measured and the fusion proteins could be purified easily in a single step by using Strep-Tactin or IMAC-Ni columns.

**Conclusions:**

This paper describes the construction and analysis of an IPTG-inducible expression vector termed P*grac100* for the high level production of intracellular recombinant proteins in *B. subtilis* and a relatively low basal expression level in *E. coli*. Based on this vector, the derivative vectors, P*grac*100-His-tag-MCS, P*grac*100-MCS-His-tag and P*grac*100-MCS-Strep-tag have been constructed.

## Background

The production of heterologous proteins in different microbial systems has revolutionized biotechnology. Most expression systems are based on an inducible promoter, and addition of the appropriate inducer leads to the production of the heterologous protein in most cases intracellularly. Microbial expression systems have been described for bacteria, yeast, filamentous fungi, and unicellular algae. All these systems have advantages and disadvantages, which have been extensively discussed [1–3].

*Escherichia coli* is the most widely used bacterial host to synthesize recombinant proteins for biochemical and functional studies. *E. coli* cells are easy to culture since they have a very short doubling time in rich media, and they are easy to manipulate genetically. Three disadvantages related to *E. coli* are: (1) low expression of some heterologous genes; (2) some heterologous proteins are insoluble and form inclusion bodies; and (3) contamination of the heterologous proteins by the endotoxin LPS [4, 5].

*Bacillus subtilis* is an attractive alternative host for heterologous protein production and engineering because of the following reasons: (1) it can secrete proteins efficiently, especially homologous proteins up to 20 g/l; (2) it is nonpathogenic; (3) and it has been granted the GRAS (generally regarded as safe) status by the American Food and Drug Administration [6–8]. The authors have developed several plasmid-based expression vectors exhibiting structural stability [9], where induction can be accomplished by addition of xylose [10], IPTG [11], glycine [12] or by a cold shock [13].

Promoter P_*spac*_ is one of the most-popular promoters used for expression of heterologous proteins in *B. subtilis*, but it is rather weak [14]. The IPTG-inducible P_*grac*_ promoter is 50 times stronger than P_*spac*_, and it has been derived from the *B. subtilis groESL* promoter and the *E. coli lac* operator [15, 16]. To further improve protein expression levels, we created a library of a second generation P_*grac*_ promoters by either introducing promoter mutations in the consensus regions resulting in stronger promoters [11] or by applying the mRNA controllable stabilizing elements (CoSE) [17]. However, enhancing the protein expression levels in *B. subtilis* also leads to higher basal expression levels in *E. coli*. In addition, some normal genes are not supposed to be harmful for *E. coli*, but it can inhibit the growth at high background expression levels, for examples ß-galactosidases, BgaB from *Geo*b*acillus stearothermophilus* and LacZ from *Escherichia coli* [18]. Therefore, expression vectors harboring promoters that control high protein production levels in *B. subtilis* after induction and allow a low basal level of expression in *E. coli* are of utmost importance. This study aims the development of expression vectors for *B. subtilis* based on a promoter that allows high inducible protein production in *B. subtilis* and relatively low basal level in *E. coli*.

## Results

### Screening for an appropriate promoter

**Fig 1:**
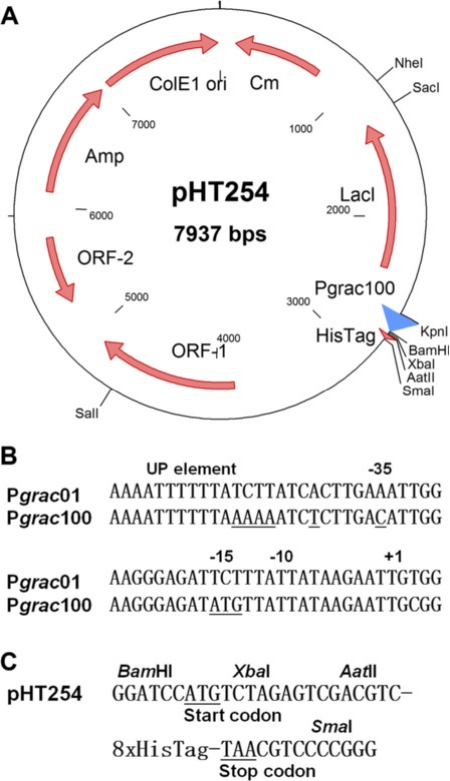
Map of the plasmid pHT254, different promoter sequences and the MCS. **a**, Map of the plasmid pHT254 with the backbone of pHT01 [16]; **b**, DNA sequences of the promoters present in P*grac*01 and P*grac*100, where the differences between these two promoters are underlined (UP element, −35, −15 regions) [11]; **c**, DNA sequence downstream of the RBS of plasmid pHT254, including the multi-cloning sites, the start codon, the His-tag and the stop codon (*Bam*HI-Start codon-*Xba*I-*Aat*II-His-tag-Stop codon/TAA-*Sma*I)

**Fig 2:**
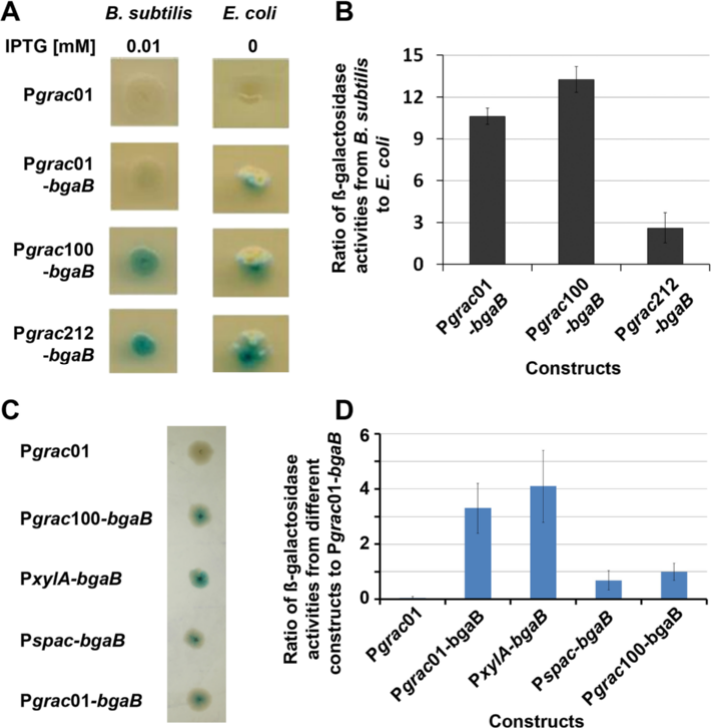
Expression of BgaB in *E. coli* OmniMax and *B. subtilis* 1012 on X-gal plates and in liquid medium. **a**, Bacterial cells containing pHT01 (P*grac*, negative control), pHT01-*bgaB* (P*grac01*-*bgaB*), pHT100 (P*grac100*-*bgaB*) and pHT212 (P*grac212*-*bgaB*) were spotted on X-gal LB agar plates containing appropriate antibiotics and 0.01 mM IPTG for *B. subtilis* and without IPTG for *E. coli* at 30 °C for 48 h. Then, pictures were taken and single colonies are shown. **b**, The bacterial cells were grown in liquid LB medium at 37 °C to the mid-logarithmic growth phase, and then induced with 0.01 mM IPTG for *B. subtilis* and kept un-induced for *E. coli*. The cells were collected after 4 h of induction, and the BgaB activities were measured. The ratio of β-galactosidase activities of the samples were calculated from induced *B. subtilis* cells and un-induced *E. coli* cells. The ratio was set as one when the BgaB activities from both *E. coli* and *B. subtilis* were identical [4, 5]. **c**, *E. coli* cells containing pHT01 (P*grac*, negative control), pHT100 (P*grac100*-*bgaB*), pHCMC04-*bgaB* (P*xylA*-*bgaB*), pHCMC05-*bgaB,* pHT01-*bgaB* (P*grac01*-*bgaB* were spotted on X-gal LB agar plates containing ampicillin. **d**, the *E. coli* cells were grown in liquid LB medium at 37 °C to the mid-logarithmic growth phase, then the growing cells collected and the BgaB activities were measured. The ratio of β-galactosidase activities of the samples were calculated from different constructs to P*grac*01-*bgaB*

Identification of a suitable inducible promoter controlling high production levels of recombinant proteins in *B. subtilis* and, at the same time, retaining relatively low basal levels in *E. coli* in the absence of the inducer is an important requirement during construction of expression vectors for *B. subtilis*. To accomplish this goal, we used the P*grac*-promoter library described [11, 17] and screened for low BgaB expression in *E. coli* by using the method described for *B. subtilis* [18]. During screening of a library of 84 different promoters, we analyzed the BgaB expression levels based on the blue color of the colonies on the X-gal plates in the absence of IPTG for *E. coli* and in the presence of 0.01 mM IPTG for *B. subtilis*. This IPTG concentration was used based on our previous results showing that IPTG and BgaB expression levels (activity) were linear for P*grac01* and promoters stronger than P*grac*01 at IPTG concentrations from 0.0025 to 0.025 mM [18]. As examples, we analyzed the three promoters P*grac*01, previously called P*grac* [11], P*grac*100 and P*grac*212. P*grac*01 is at least 50-times stronger than P*spac* [14] based on BgaB activities and allowed BgaB protein accumulation up to 9.1 % of total the cellular proteins [11, 15]. P*grac*212 is structurally similar to P*grac*01 containing modifications at the controllable stabilizing element (CoSE) – the region from +1 up to the RBS – resulting in BgaB levels within the same range as compared to P*grac*100 [17]. P*grac*100 is different from P*grac*01 at the UP-element (−44-TCTTATCT--37 - > −44-AAAAATCT--37), the −35 motif (TTGAAA - > TTGACA), and the −15 region (−16-TCT—14 - > −16-ATG--14) (Fig. 1a). The negative control plasmid, P*grac*01 without the *bgaB* gene exhibited white colonies for both *B. subtilis* and *E. coli* on X-gal plates (Fig. 2a). When the strength of the 84 different promoters was analyzed on X-gal plates, P*grac*100-*bgaB* and P*grac*212-*bgaB* exhibited a stronger blue color in comparison to P*grac*01-*bgaB* in *B. subtilis* in the presence of IPTG (Fig. 2a). When these plasmids were analyzed in *E. coli* in the absence of the inducer, P*grac*212-*bgaB* exhibited the strongest blue color, followed by P*grac*100-*bgaB* and P*grac*01-*bgaB* (Fig. 2a). *E. coli* colonies sometimes showed that only a part of the colonies were blue. However, this is not an indication that the plasmids were structurally unstable. The stability of the plasmid backbone derived from pHT01-*bgaB* was confirmed previously [16]. Calculation of the grey values from these colonies confirmed the result observed by eyes. By screening the 84-promoter library, P*grac*100 appeared to be the most appropriate one that met the criteria for an optimal inducible promoter. It has a relatively low background level in *E. coli* and a high inducible expression level in *B. subtilis*.

### Choice of promoter P*grac*100

To have a clearer picture of the P*grac*100 promoter, we measured the ß-galactosidase (BgaB) activities of potential promoter candidates from cells grown in liquid LB medium for both *E. coli* in the absence of IPTG and *B. subtilis* after addition of the inducer. The ratios of ß-galactosidase activities obtained with *B. subtilis* and *E. coli* were calculated, representing the promoter strengths in both species. High activity in *B. subtilis* and high ratio numbers indicate a better promoter. We used P*grac*01 (formerly P*grac*) as the reference. The *B. subtilis* and *E. coli* cells containing pHT01 (P*grac* without *bgaB*, negative control) do not produce detectable ß-galactosidase activity. As an example, Fig. 2b shows that P*grac*100-*bgaB* has a higher ratio than P*grac*01-*bgaB* and more than three times higher ratio than P*grac*212-*bgaB*. In addition, P*grac*100-*bgaB* is about 9.2 times higher than P*grac*01-*bgaB* after induction at 0.01 mM IPTG (Table 1). When these two values were compared with those obtained with other promoters in our library, the BgaB activities indicated that P*grac*100 is the most appropriate candidate that controls high production levels of recombinant proteins in *B. subtilis* and at the same time maintains a relative low background expression in *E. coli* (data not shown).

**Table 1:** Expression of *bgaB* and *gfp +* under control of P*grac*01 and P*grac*100

	*E. coli*	*B. subtilis*			
IPTG concentration	0 mM	0 mM	0.001 mM	0.01 mM	0.1 mM
P*grac*01-*bgaB*					
0 h	7.7 ± 2.4	6.4 ± 1.4			
4 h	5.3 ± 1.1	38.9 ± 5.8	50 ± 12	53 ± 15	257 ± 31
P*grac*100-*bgaB*					
0 h	26 ± 9	75 ± 9			
4 h	37 ± 5.7	222 ± 68	343 ± 78	489 ± 119	817 ± 79
P*grac*01-*gfp*					
0 h	68 ± 4.7	5 ± 0.7			
4 h	47 ± 1.5	8 ± 0.5	7.0 ± 0.5	21 ± 0.6	190 ± 8
P*grac*100-*gfp*					
0 h	29 ± 0.7	73 ± 2.3			
4 h	37 ± 2.6	63 ± 2.6	106 ± 9.4	568 ± 117	1554 ± 65

The data for BgaB and GFP activity presented have been obtained with pHT01-*bgaB* (P*grac*01-*bgaB*), pHT10-*gfp +* (P*grac*01-*gfp*) pHT212 (P*grac*212-*bgaB*) and pHT100-*gfp* (P*grac*100-*gfp*). BgaB activity is shown in Miller units while GFP indicated as activity is relative fluorescence unit (RFU). All experiments were carried out from at least three different colonies, and standard errors were calculated

The results in Table 1 also showed that P*grac*100-*bgaB* seems to be characterized from high basal expression (222 ± 68 units) in *B. subtilis*. If low basal expression in *E.coli* is important to facilitate the cloning of toxic genes, the presence of basal expression in *B. subtilis* could make difficult the plasmid transformation. However, it also indicated that we could use P*grac*100 promoter for high production levels of recombinant protein at low concentration of IPTG inducer. If we consider low background expression levels in *E. coli* and *B. subtilis*, selection of P*grac*01 [16] could be an option.

In comparison with other systems, we transformed P*xylA*-*bgaB* (pHCMC04-*bgaB*) and P*spac*-*bgaB* (pHCMC05-*bgaB*) [9] into *E. coli* and spread transformants on X-gal plates, and the *E. coli* colonies developed blue color. Colonies of P*spac*-*bgaB* were within the same range as those from P*grac*01-*bgaB*, while colonies from P*xylA*-*bgaB* were deeper blue than the others (Fig. 2c). When the *E. coli* cells were growth in liquid LB medium in the absence of the inducers, BgaB activities from P*spac*-*bgaB* were equal to that of P*grac*01-*bgaB*, while those from P*xylA-bgaB* were within the same range as those from P*grac*100-*bgaB* (Fig. 2d). In *B. subtilis*, the BgaB expression levels of the two constructs, P*spac*-*bgaB* and P*xylA*-*bgaB* in the presence of inducers were within the same range [9] and 50 times lower than P*grac*01-*bgaB* [15, 16]. Though P*spac* expressed lower basal levels than and P*xylA* as high as P*grac100* in *E. coli*, the expression levels in *B. subtilis* was also very low in the presence of inducer. Therefore, these promoters are not appropriate to be used for over-production of recombinant proteins in *B. subtilis*.

### Important factors of P*grac*100 in controlling GFP expression

Though BgaB is a popular reporter protein for *B. subtilis*, it has heterogeneous properties in *E. coli* [19]. In order to confirm the properties of P*grac*100, we replaced the *bgaB* gene by *gfp +* (pHT100-*gfp*; Table 2) and analyzed for GFP expression. The background expression level of GFP from P*grac*100-*gfp* in *E. coli* is 37 RFU (Relative Fluorescence Unit), while that of P*grac*01-*gfp* is 68 RFU. In addition, the ratio of GFP activities of the background expression level in *E. coli* and in *B. subtilis* cells induced with 0.01 mM IPTG in for P*grac*100-*gfp* turned out to be 15.3 while that of P*grac*01-*gfp* was 0.5 (Table 1) and that of P*grac*212-*gfp* 0.2. These results clearly confirm that the promoter P*grac*100 is able to tightly control protein expression in *E. coli* at the same range as compared with P*grac*01.

**Table 2:** Bacterial strains, plasmids and oligonucleotides used in this study

Bacterial strains	Genotype	Source/reference
*E. coli* OmniMAX	*mc*r*A* Δ*(mrr hs*d*RMS*-*mc*r*BC*); resistant to T1 and T5 phage; used for cloning	Invitrogen
*B. subtilis* 1012	*leuA8 metB5 trpC2 hsrM1*	[22]
**Plasmid**	**Description**	**Source/reference**
pHCMC04-*bgaB*	P*xylA-bgaB*	[9]
pHCMC05-*bgaB*	P*spac*-*bgaB*	[9]
pHT01	P*grac01 (previously called Pgrac)*	[16]
pHT01-*bgaB*	P*grac01*-*bgaB*	[16]
pHT10-*gfp+*	P*grac*-*gfp-Strep*	[16]
pHT100	P*grac*100-*bgaB*	[11]
pHT100-*gfp+*	Pgrac100-*gfp*	This study
pHT212	P*grac*212-*bgaB*	[17]
pHT253	P*grac*100-8xHis-MCS (Start codon-His-tag-*Bam*HI-*Xba*I-*Aat*II-*Sma*I)	This study
pHT254	P*grac*100-MCS-His (*Bam*HI-start codon-*Xba*I-*Aat*II-His-tag-stop codon/TAA)	This study
pHT255	P*grac*100-MCS-Strep (*Bam*HI-start codon-*Xba*I-*Aat*II-Strep-tag-stop codon/TAA)	This study
pHT1169	P*grac*100-His-*gfp*	This study
pHT1170	P*grac*100-*gfp*-His	From Nguyen H. N
pHT1171	P*grac*100-*gfp*-Strep	This study
pHT1178	P*grac*100-8xHis-*bgaB*	From Nguyen H. N
pHT1179	P*grac*100-*bgaB*-His	This study
pHT1180	P*grac*100-*bgaB*-Strep	This study
^**a**^ **Oligonucleotide**	**Sequence 5′ → 3′**	**Used for**
ON301F	GATCTATGGAAGCTCATCACCATCACCATCACCATCACGGATCCATGTCTAGAGTCGACGT	pHT253
ON302R	CGACTCTAGACATGGATCCGTGATGGTGATGGTGATGGTGATGAGCTTCCATA	pHT253
ON303F	GATCCATGTCTAGAGTCGACGTCGCTCATCACCATCACCATCACCATCACTAACGT	pHT254
ON304R	TAGTGATGGTGATGGTGATGGTGATGAGCGACGTCGACTCTAGACATG	pHT254
ON305F	GATCCATGTCTAGAGTCGACGTCGCTTGGAGCCATCCGCAATTTGAAAAATAACGT	pHT255
ON306R	TATTTTTCAAATTGCGGATGGCTCCAAGCGACGTCGACTCTAGACATG	pHT255
ON941	AAAGGAGGAAGGATCCATGAATGTGTTATC	pHT1179 and pHT1180
ON1250	CTGCCCCGGGGACGTCAACCTTCCCGGCTTCATCATGC	pHT1179 and pHT1180
ON1277	AAAGGAGGAAGGATCCATGGCTAGCAAAGGAGAAGAACT	pHT1169 and pHT1171
ON1278	GGCCATGACGTCTTTGTAAAGCTCATCCATGCCATGTGT	pHT1171
ON1279	CCAGGTCTCAGATCTATGGCTAGCAAAGGAGAAGAACT	pHT100-*gfp+*
ON1280	GGCCATGACGTCTTATTTGTAAAGCTCATCCATGCCATGTGT	pHT100-*gfp +* and pHT1169

^a^The restriction sites used for plasmid construction are underlined. P*grac*01 (another name is P*grac*), P*grac*100 and P*grac*212 are the name of different promoters; MCS, multi-cloning site; Strep, Strep-tag; His, His-tag

The expression levels of GFP of P*grac*100-*gfp* increased after addition of IPTG and reached up to 568 RFU at 0.01 mM IPTG, 27-fold higher than that of P*grac*01-*gfp* (Table 1) and 4.7-fold higher than that of P*grac*212-*gfp*. In addition, we calculated the induction factor and the ratio of the activities of induced and un-induced samples. P*grac*100 exhibited an induction factor of 9 at 0.01 mM IPTG and of 25 at 0.1 mM IPTG (Table 1), while those of P*grac01* were 2.6 and 24.7, respectively (Table 1) and those of P*grac212* 6.3 and 77 (data not shown). Similar results using BgaB as reporter were also observed for P*grac100* (Table 1). The substantial differences in protein expression levels between BgaB and GFP might be because they come from two different organisms, BgaB from *G. stearothermophilus* and GFP from *Aequorea victoria*, and the sequences of the genes might influence the transcription and/or translation efficiency in *E. coli* and *B. subtilis*. These results demonstrate that P*grac100* not only tightly controls the background expression level in *E. coli*, but also allowed high protein production levels at low IPTG concentrations. In summary, promoter P*grac100* is an excellent choice for the construction of inducible expression vectors for *B. subtilis*.

### Construction of basic expression vectors

The above result demonstrated that promoter P*grac*100 allowed high protein production levels in *B. subtilis* and low background expression levels in *E. coli* by using two reporter proteins, BgaB and GFP. To generate tagging expression vectors, we removed *bgaB* from pHT100 [11] and added the DNA fragments containing start codon-His-tag-*Bam*HI-*Xba*I-*Aat*II-*Sma*I, *Bam*HI-start codon-*Xba*I-*Aat*II-His-tag-stop codon/TAA or *Bam*HI-start codon-*Xba*I-*Aat*II-Strep-tag-stop codon/TAA, resulting pHT253, pHT254, and pHT255, respectively (Table 2). Fig. 1c shows the DNA sequence of the multi-cloning site, the His-tag, the start and the stop codon from pHT254. The other plasmids were adapted appropriately to meet these requirements. The full sequences of these three plasmids were similar to pHT01 [16] except for the promoter regions and the multi-cloning sites with different tags. The map of plasmid pHT254 is shown in Fig. 1a. Fig. 2b indicates the differences between promoter P*grac*01 and P*grac*100 at the UP element and the −35 and −15 regions. Target genes can be introduced using restriction enzymes *Bam*HI, *Xba*I, *Aat*II or *Sma*I as fusions or non-fusions with either a His-tag or a Strep-tag at the N- or C-terminus.

### Evaluation of the expression vectors in *B. subtilis*

**Fig 3:**
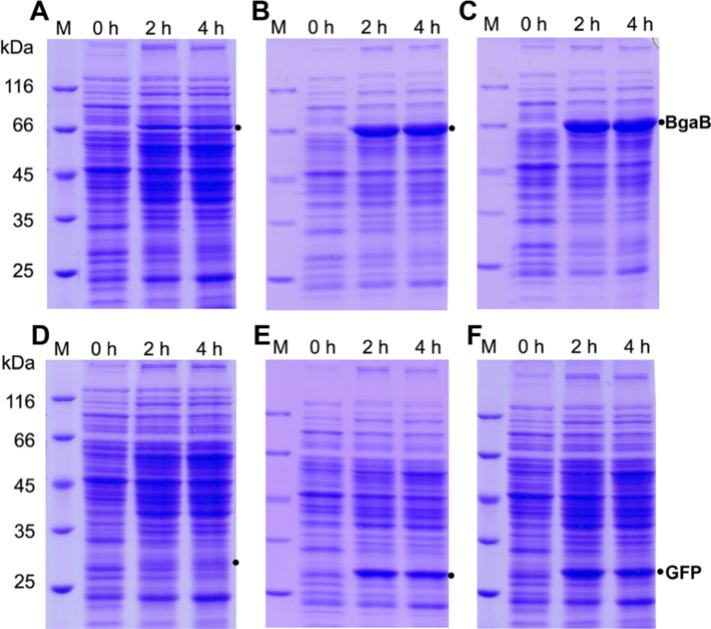
Expression of *gfp +* and *bgaB* fused to a His- or a Strep-tag. *B. subtilis* 1012 carrying (**a**) pHT253-*bgaB* (His-tag-BgaB), (**b**) pHT254-*bgaB* (BgaB-His-tag), (**c**) pHT255-*bgaB* (BgaB-Strep-tag), (**d**) pHT253-*gfp* (His-tag-GFP), (**e**) pHT254-*gfp* (GFP-His-tag), (**f**) pHT255-*gfp* (GFP-Strep-tag) were grown in LB medium to mid-log, and production of the recombinant proteins was induced by addition of 0.1 mM IPTG. Aliquots were taken before addition of IPTG and 2 and 4 h later. Cells were lysed by lysozyme, and aliquots corresponding to an OD_600_ of 0.13 were analyzed by SDS-PAGE (lane 0 h, before induction; lanes 2 h and 4 h, 2 and 4 h after induction). Black dots indicate the positions of BgaB or GFP, respectively

To evaluate the basic expression vectors pHT253, pHT254, and pHT255, we introduced *gfp +* or *bgaB* as translational fusions with 8xHis- or Strep-tags resulting in pHT1169 (8xHis-*gfp*), pHT1170 (*gfp*-8xHis), pHT1171 (*gfp*-Strep) and pHT1178 (8xHis-*bgaB*), pHT1179 (*bgaB*-8xHis) and pHT1180 (*bgaB*-Strep) (Table 2). Plasmids containing the Strep-tag at the N-terminus fused with the reporters were also constructed, but the production levels were very low (data not shown). Fig. 3 shows expression of BgaB and of GFP fused to the His- or Strep-tag under control of P*grac*100 after induction with 0.1 mM IPTG. The His-tag at the N-terminus in plasmid pHT253 drastically reduced the expression levels of BgaB, reaching 6.2 % of the total cellular proteins (Fig. 3a) and GFP (Fig. 3d) compared to the fusions at the C-terminus and in the absence of any tag. The expression levels of BgaB and GFP in these constructs are equal to those in pHT01-*bgaB* (P*grac*01-*bgaB*) and pHT10-*gfp* (P*grac*01-*gfp*) [16] in terms of their activities. These results indicate the expression levels of P*grac*100 with the His-tag at the N-terminus are comparable to P*grac* synthesizing BgaB and GFP.

The fusions, BgaB-His (Fig. 3b), BgaB-Strep (Fig. 3c), GFP-His (Fig. 3e) and GFP-Strep (Fig. 3f) are produced at levels comparable to those without a purification tag, BgaB (from pHT100-*bgaB*) and GFP (from pHT100-*gfp*) deduced from SDS-PAGE gels. The BgaB expression levels could reach up to 30 % of total cellular proteins [11], while the tagged versions accumulated 24 % using 0.1 mM IPTG (Fig. 3b and c) and up to 30 % using 1 mM IPTG. Similarly, the untagged and the C-tagged versions of GFP could be produced at 15 % of total cellular proteins on the average (Figure 3e and f). However, the expression levels of the fusions at low concentrations of IPTG were lower than the untagged constructs. Besides in *B. subtilis* 1012, we also checked the expression in *B. subtilis* WB800N [20], a derivative of WB800 [21], a derivative of strain 168. The expression levels in WB800N were similar to those in 1012 (data not shown). These results indicate that the expression vectors pHT253, pHT254, pHT255 could be used for overproduction of recombinant proteins to high levels in different *B. subtilis* strains.

**Fig 4:**
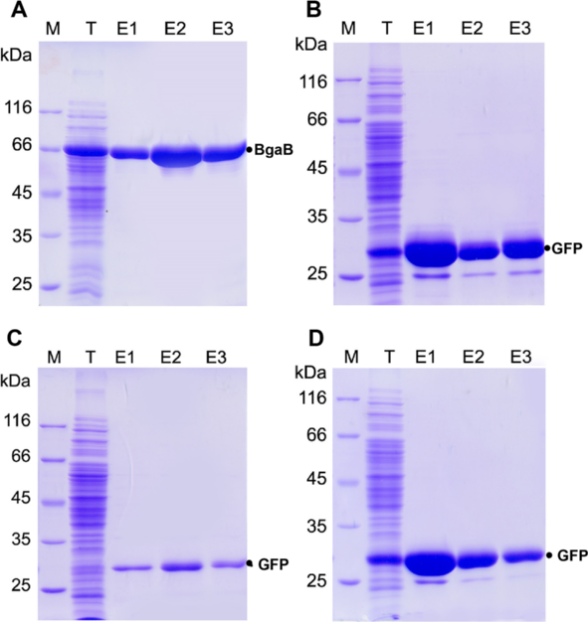
Overexpression and affinity purification of proteins fused to a His- or Strep-tag. *B. subtilis* 1012 carrying (**a**) pHT254-*bgaB* (BgaB-His-tag), (**b**) pHT254-*gfp* (GFP-His-tag), (**c**) pHT253-*gfp* (His-tag-GFP) and (**d**) pHT255-*gfp* (GFP-Strep-tag) were grown in LB medium to mid-log phase, and production of the recombinant proteins was induced by the addition of 0.1 mM IPTG. Cells were lysed, and aliquots were analyzed by SDS-PAGE (lane T, total cellular protein). The cellular extracts were applied to appropriate affinity columns, washed extensively and the bound protein was eluted as described under Materials and Methods. E1, E2 and E3 indicate the first, the second and the third elution step, respectively

To enhance the use of the affinity tags, His- and Strep-tags were introduced in the primary expression vectors pHT253 (His-tag), pHT254 (His-tag) and pHT255 (Strep-tag). The His-tag is widely used in conjunction with metal chelate resins and the Strep-tag (sequence: WSHPQFEK) is an alternative purification tag that binds at high specificity and affinity to streptavidin. Both tags allow a one-step purification of recombinant proteins using affinity chromatography. These tags were already successfully used in our earlier expression vectors pHT08, pHT09 and pHT24 [16] based on P*grac*01. For this purpose, the *B. subtilis* 1012 cells containing pHT254-*bgaB* (BgaB-His, Fig. 4a), pHT254-*gfp* (GFP-His, Fig. 4b), pHT253-*gfp* (His-GFP, Fig. 4c), and pHT255-*gfp* (GFP-Strep, Fig. 4d) were induced with 0.1 mM IPTG for expression of the tagged genes. The clear lysates of the disrupted cells were applied to the Ni-NTA or Strep-Tactin Spin columns according to the instructions provided. The fusion proteins were eluted in three fractions (E1, E2 and E3) and compared with the lysate sample (T) as shown in Fig. 4. While the production of the fusion proteins were high for BgaB-His (Fig. 4a, lane T), GFP-His (Fig. 4b, lane T) and GFP-Strep (Fig. 4d, lane T), the expression of His-GFP (Fig. 4c, lane T) was rather low. All of the fusion proteins could be quickly purified. These results demonstrate that the fusion proteins with either a His- or a Strep-tag could be purified to near homogeneity in a single step.

## Conclusions

We show that the artificial promoter P*grac*100 could be used for the construction of His- or Strep-tagged versions, pHT253, pHT254 and pHT255 for *B. subtilis*. These three new expression vectors provide two advantages: (i) allowing high production levels of recombinant proteins in *B. subtilis* after induction and (ii) maintaining relatively low background expression levels in *E. coli*.

## Methods

### Bacterial strains, plasmids and growth conditions

*E. coli* strain OmniMAX (Invitrogen) was used as the recipient in all cloning experiments and to determine the expression levels. *B. subtilis* strains, 1012 [22] and WB800N [20] were used to analyze expression of the *bgaB* and *gfp+* genes. A list of the plasmids and oligonucleotides used in this study is shown in Table 2. Cells were routinely grown in Luria broth (LB) at 37 °C under shaking at 200 rpm. Antibiotics were added where appropriate (ampicillin at 100 μg/mL for *E. coli* and chloramphenicol at 10 μg/mL for *B. subtilis*).

#### Construction of plasmids

The plasmid pHT100 [11] carrying promoter P*grac100* fused to the reporter gene *bgaB* was used as backbone. To generate the primary expression vectors, we removed the *bgaB* gene and inserted DNA sequences coding for a His- or a Strep-tag either to the N- or to the C-terminus. Three pairs of complementary oligonucleotides (ON), ON301F and ON302R, ON303F and ON304R, ON305F and ON306R were used for these purposes. The complementary mixtures were ligated with the *Bam*HI- and *Aat*II-treated pHT100 resulting in pHT253, pHT254 and pHT255. These vectors contain start codon-His-tag-*Bam*HI-*Xba*I-*Aat*II-*Sma*I (pHT253), *Bam*HI-start codon-*Xba*I-*Aat*II-His-tag-stop codon/TAA (pHT254) or *Bam*HI-start codon-*Xba*I-*Aat*II-Strep-tag-stop codon/TAA (pHT255). To construct pHT100-*gfp +* (GFP+), pHT1169 (His-tag-GFP+) and pHT1171 (GFP + −Strep-tag), we amplified the *gfp +* gene using the primer pairs, ON1279 and ON1280 for pHT100-*gfp+*, ON1277and ON1280 for pHT1169, and ON1277 and ON1278 for pHT1171 with pHT10-*gfp +* [16]. The *Bgl*II- or *Bam*HI- and *Aat*II-treated PCR products were introduced into pHT100, pHT253 or pHT255 at *Bam*HI and *Aat*II, resulting in pHT100-*gfp+*, pHT1169 and pHT1171, respectively. To construct pHT1179 (*bgaB*-His-tag) and pHT1180 (*bgaB*-Strep-tag), we amplified the *bgaB* gene using primers ON941 and ON1250 with pNDH33-*bgaB* [15] as template. The *Bam*HI- and *Aat*II-treated PCR products were ligated into pHT254 and pHT255 at their *Bam*HI and *Aat*II sites resulting in pHT1179 and pHT1180, respectively.

#### Measurement of the BgaB and GFP production levels in *E. coli* and in *B. subtilis*

Three colonies were cultured in 0.5 ml LB medium containing the appropriate antibiotic in a 96 well-block (Eppendorf block) and shaken overnight at 200 rpm at room temperature (25 °C). The pre-culture of each clone (75 μl) was transferred to 3 ml LB medium containing the appropriate antibiotic in a 24-well-block. The block was incubated at 37 °C with shaking at 200 rpm. When the OD_600_ of the culture reached 0.6 – 1, the cells were induced by addition of IPTG at final concentration of 0 mM, 0.001 mM, 0.01 mM and 0.1 mM. The cells were harvested after 2 or 4 h of induction. The cells were collected in Eppendorf tubes at an OD_600_ of 2.5 after centrifugation. Samples were prepared for activity measurements or SDS-PAGE. The cells were lysed by lysozyme and sample buffer was added to 150 μl, and 8 μl each were applied to SDS-PAGE. ß-galactosidase activities were measured as described [23]. For *E. coli*, the GFP cells were re-suspended in 300 μl BPS, 12 μl chloroform, and 6 μl SDS 0.1 % were added followed by shaking for 1 h. For *B. subtilis*, the GFP cells were lysed in 300 μl PBS containing 1 mg/ml lysozyme and incubated at 37 °C for 2 h. The samples were centrifuged at 10 000 rpm for 5 min and used for determination of the activities. GFP activities were measured by using a Synergy HT Multi-mode Microplate Reader and 384 W plate (Black) with an excitation wavelength at 485 (+/−20) nm and an emission wavelength at 520 (+/−20) nm. The experiment was carried with at least three different colonies, and standard errors were calculated.

#### Affinity purification of the fusion proteins

*B. subtilis* 1012 carrying different plasmids were grown in LB medium to mid-log phase, and production of the recombinant proteins was induced by addition of 0.1 mM IPTG. The cells were collected by centrifugation and re-suspended in the desired buffers with lysozyme (0.25 mg/ml) and disrupted by sonification. For His-tag fusion proteins, the protocol with recommended buffers for Ni-NTA Spin Columns (Qiagen) was applied, in which the washing buffer contain 40 mM imidazole. For Strep-tag fusion proteins, the Strep-Tactin Spin column kit (IBA) was used.
